# Autologous Epidermal Grafting Using a Novel Negative Pressure Epidermal Harvesting System in a Case of Stable Vitiligo

**DOI:** 10.7759/cureus.881

**Published:** 2016-11-15

**Authors:** Aarthi Krishna, Vanathi Thirunavukkarasu, Paru Priyadarshini Navaneetha Krishnan, Nithya Gayathri Devi Danasekaran, Ratnavel Rajendrabose

**Affiliations:** 1 Department of Cosmetology, Government Stanley Medical College and Hospital

**Keywords:** vitiligo, epidermal grafting, negative pressure, repigmentation

## Abstract

Vitiligo is a common pigmentary disorder of the skin with a great amount of social stigma attached to it. Though various medical modalities are available for the treatment of stable vitiligo, surgical modality remains the treatment of choice for stable and localized vitiligo. The surgical options range from simple punch grafting to the recent epidermal harvesting methods using a negative pressure unit. Although successful use of multiple methods of epidermal grafting has been reported, most of them are cumbersome and time-consuming. The new automated epidermal harvesting system now commercially available involves a tool that applies both heat and suction concurrently to normal skin to induce epidermal micrografts. Hence it serves as a safe, quick and cost-effective method without anesthesia, with a very minimal downtime for healing and requires an optimal expertise. The duration of repigmentation seems to be faster and more uniform compared to other procedures. We would like to share our experience with the negative pressure epidermal harvesting method in a patient with stable vitiligo.

## Introduction

Vitiligo is a common acquired skin disorder characterized by depigmentation of the skin. All forms of treatment for vitiligo aim at providing repigmentation either by stimulating or repopulating the melanocytes or targeting the immune mechanisms associated with the etiopathogenesis. Surgical treatment of vitiligo is beneficial especially in stable vitiligo and where the patients are recalcitrant to medical modalities of treatment. The various surgical measures aim to stimulate melanocytes from the normal peripheral skin and hair follicles by therapeutic wounding, repopulation of the depleted melanocytes by grafting or cell suspension techniques, introduction of artificial pigments into the lesions by tattooing and removal of the depigmented areas forever by excision and primary closure. Skin grafting in vitiligo consists of tissue or cellular grafting [1-2]. Tissue grafts comprise of miniature punch grafting, split skin grafting and suction blister grafting. Cellular grafts consist of transferring melanocyte suspension obtained by culture or non-culture methods. Newer methods in skin grafting like the Autologous Negative Pressure Epidermal Harvesting (ANPEH) are based on the modified technique of suction blister grafting, using a sustained vacuum pressure and heat, which results in the formation of microdomes of the epidermis. Compared to other methods of autologous skin grafting, the ANPEH procedure has the benefits of reduced downtime for patients, rapid onset of pigmentation and better color match. Here we report the successful treatment of a stable vitiligo using the ANPEH system.

## Case presentation

**Figure 1:**
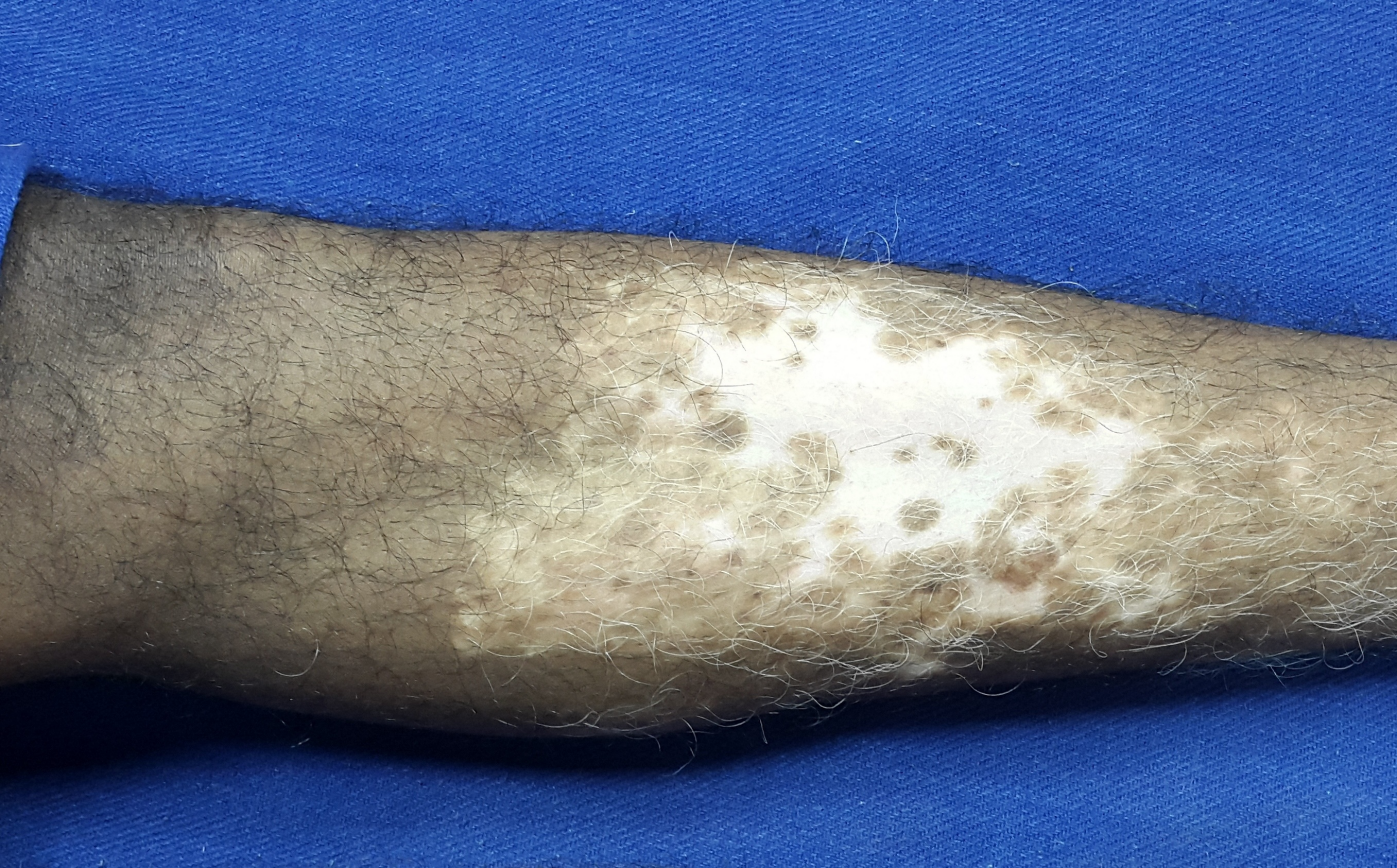
Pre-procedure photograph showing the vitiliginous plaque over the shin of the left leg.

A 26-year-old male, who was a counter salesman by occupation, presented to our out-patient department with a depigmented patch over the shin of his left leg. The lesion had started five years back as a small area of hypopigmentation, which gradually increased in size over a period of three years to attain the present size. It has been stable since then with no further increase in size or no newer areas of depigmentation elsewhere in the body. He had been applying topical steroids and tacrolimus 0.1% ointment for the past three years with not much improvement. His quality of life was greatly affected by the illness as he was facing stigmatization at his workplace and among his relatives. History suggestive of keloidal tendency, bleeding diathesis, other major systemic illnesses and family history of similar illness were ruled out. On examination, there was a well- to ill-defined hypopigmented to depigmented plaque of size 15*9 cm over the middle 1/3rd of the anterior aspect of the left leg with leukotrichia at the center and few areas of repigmentation. Other parts of the skin and mucosa were normal. His baseline investigations and coagulation profile were within normal limits. Hepatitis and retroviral serology were negative. A pre-procedure photograph was taken to assess the treatment outcome (Figure 1).

**Figure 2:**
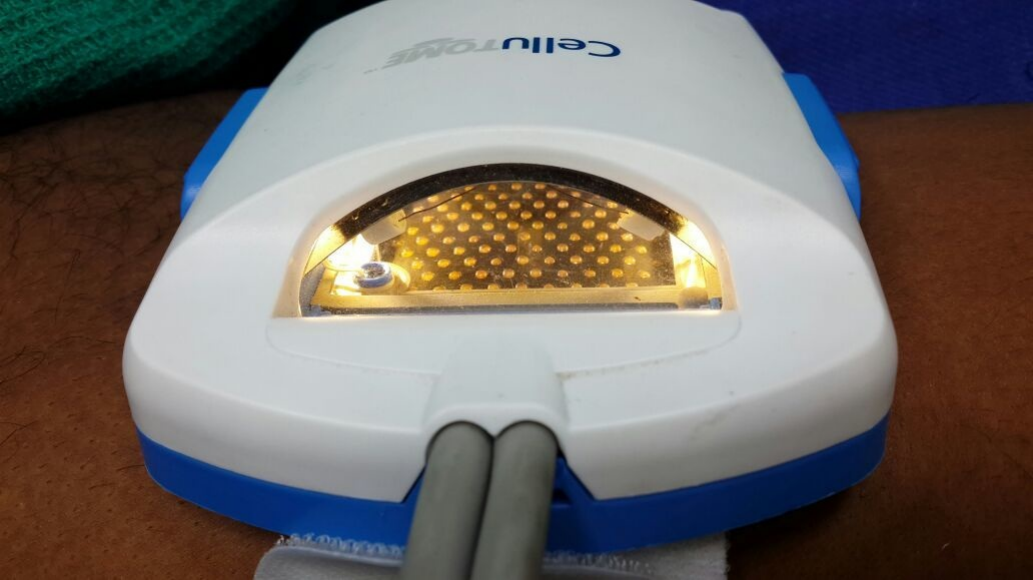
Visualization of the formation of microdomes through the view window.

**Figure 3:**
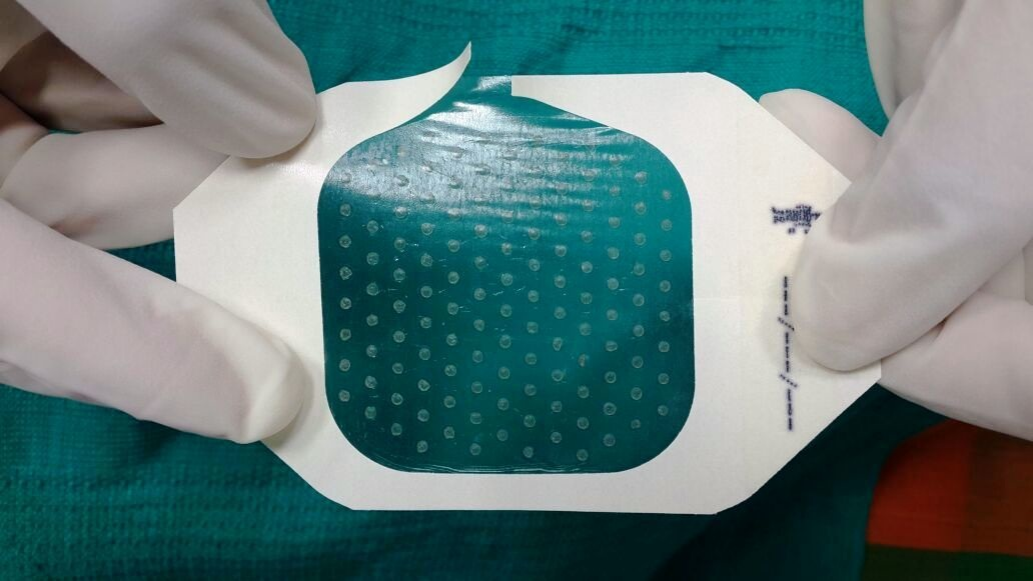
Image showing the epidermal grafts adhered to the Tegaderm dressing.

**Figure 4:**
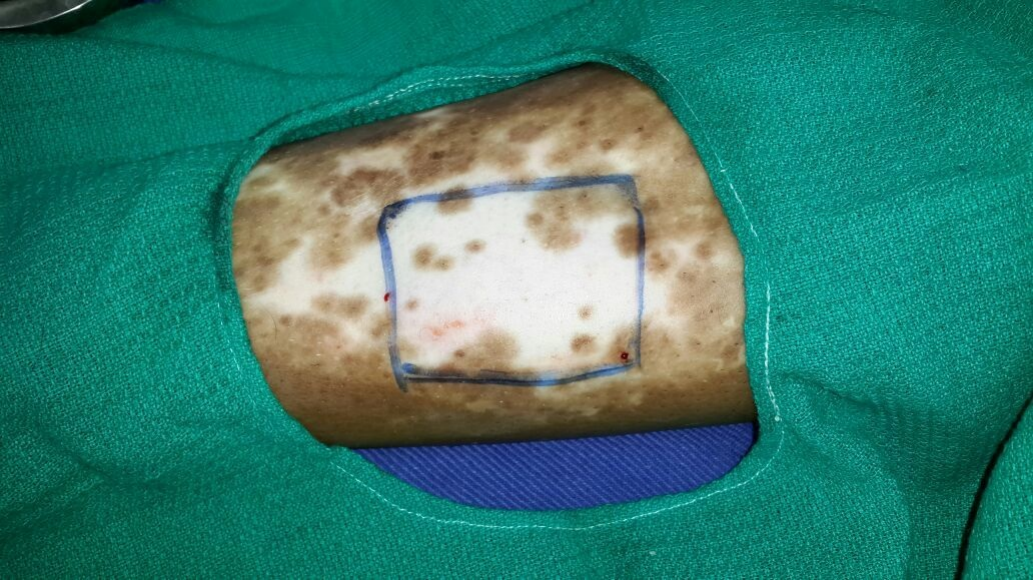
The recipient site is cleansed, draped, and marked.

**Figure 5:**
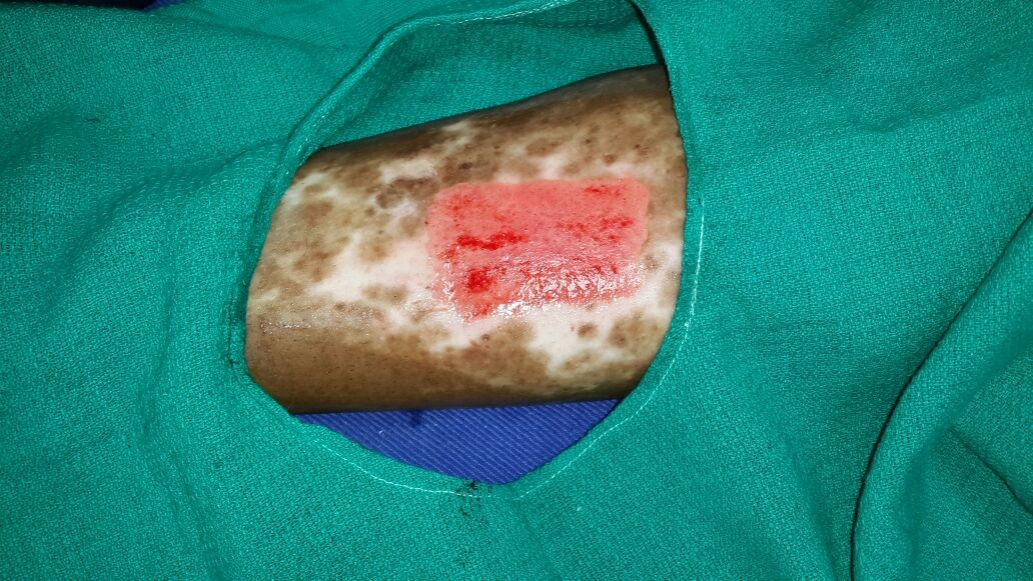
The recipient site is dermabraded using a micro-motor with diamond fraise until pinpoint bleeding.

**Figure 6:**
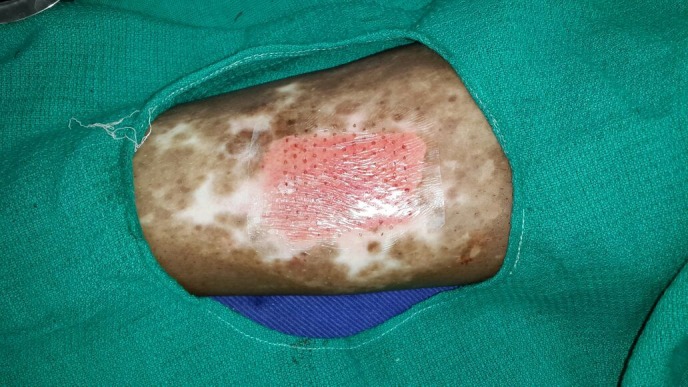
The Tegaderm dressing with the attached micrografts is placed over the recipient site.

With written and informed consent, the patient was subjected to the ANPEH procedure. The donor site chosen was the anterolateral aspect of the right thigh for better patient convenience and color match. The area was cleaned and draped with aseptic precautions. No anesthesia was given. The harvester (Cellutome™ Harvester, Acelity, Texas, USA), (a disposable component with a cutting blade), was placed with the curved blade side upwards and secured with the Velcro strap provided. The vacuum head (a reusable component of the system that delivers negative pressure and warming from the control unit to the harvester) was then applied to the harvester ensuring a snug fit. The power of the control unit was switched on, and the skin was heated at 37°C to 41°C and a negative pressure of -400 mm Hg to -500 mm Hg was rendered and the process of formation of microdomes of epidermis began. The microdomes were visualized through the view window (Figure 2). At the end of 30 minutes, round well-formed microdomes were visualized and the unit was turned off. The vacuum head was removed and a Tegaderm™ (3M, Minnesota, USA) dressing (with perforations made using an 18 G needle for exudation of serum during the postoperative days), was inserted and the blade was activated by lowering the handle. The dressing was then gently removed with the adhered microdomes (Figure 3). The patient experienced a very minimal and bearable discomfort during the procedure. The donor site was dressed with sterile gauze. The recipient site was cleansed, draped and marked (Figure 4). Under local anesthesia, the recipient site was dermabraded using a micromotor with diamond fraise (Figure 5). The Tegaderm dressing with the grafts embedded was spread uniformly over the denuded recipient site (Figure 6). The area was in turn dressed with an absorbable sterile pad which was secured in place using a dynaplast. The limb was kept immobile for 30 minutes and the patient was sent home with oral antibiotics and analgesics. He was advised to avoid strenuous activities for a week.

**Figure 7:**
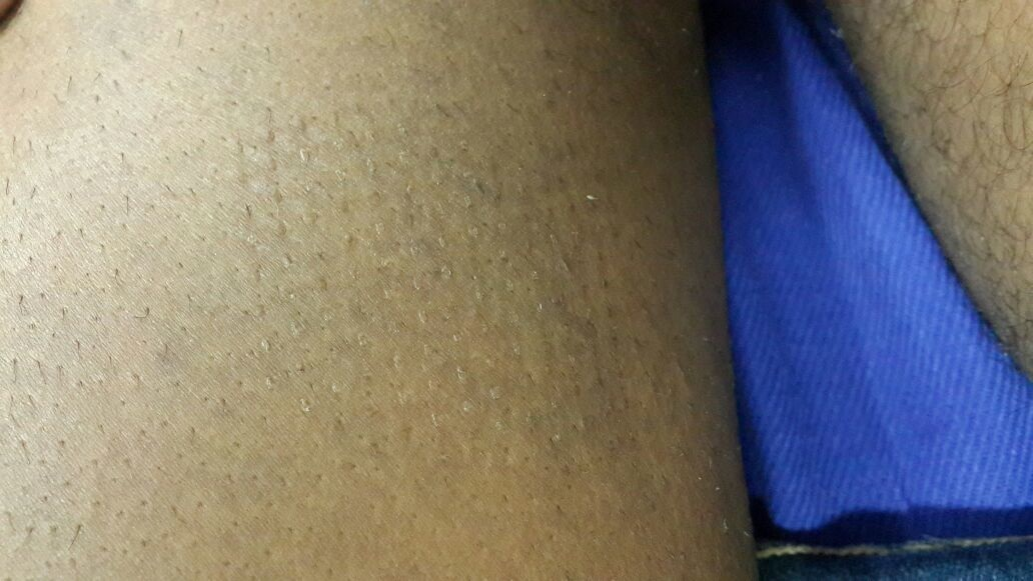
Image showing complete healing of the donor site.

**Figure 8:**
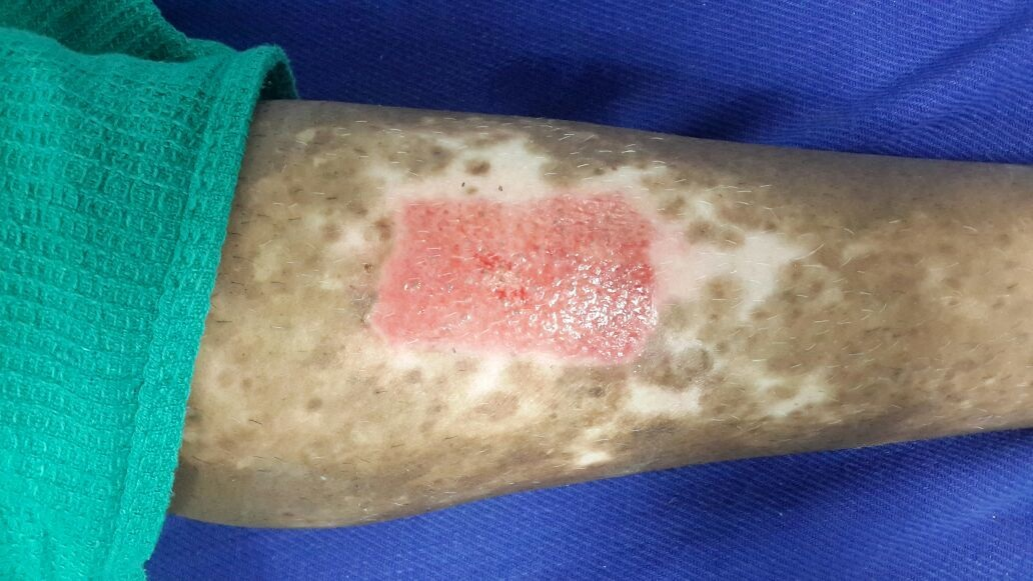
The recipient site at the end of one week showing grafts falling off.

**Figure 9:**
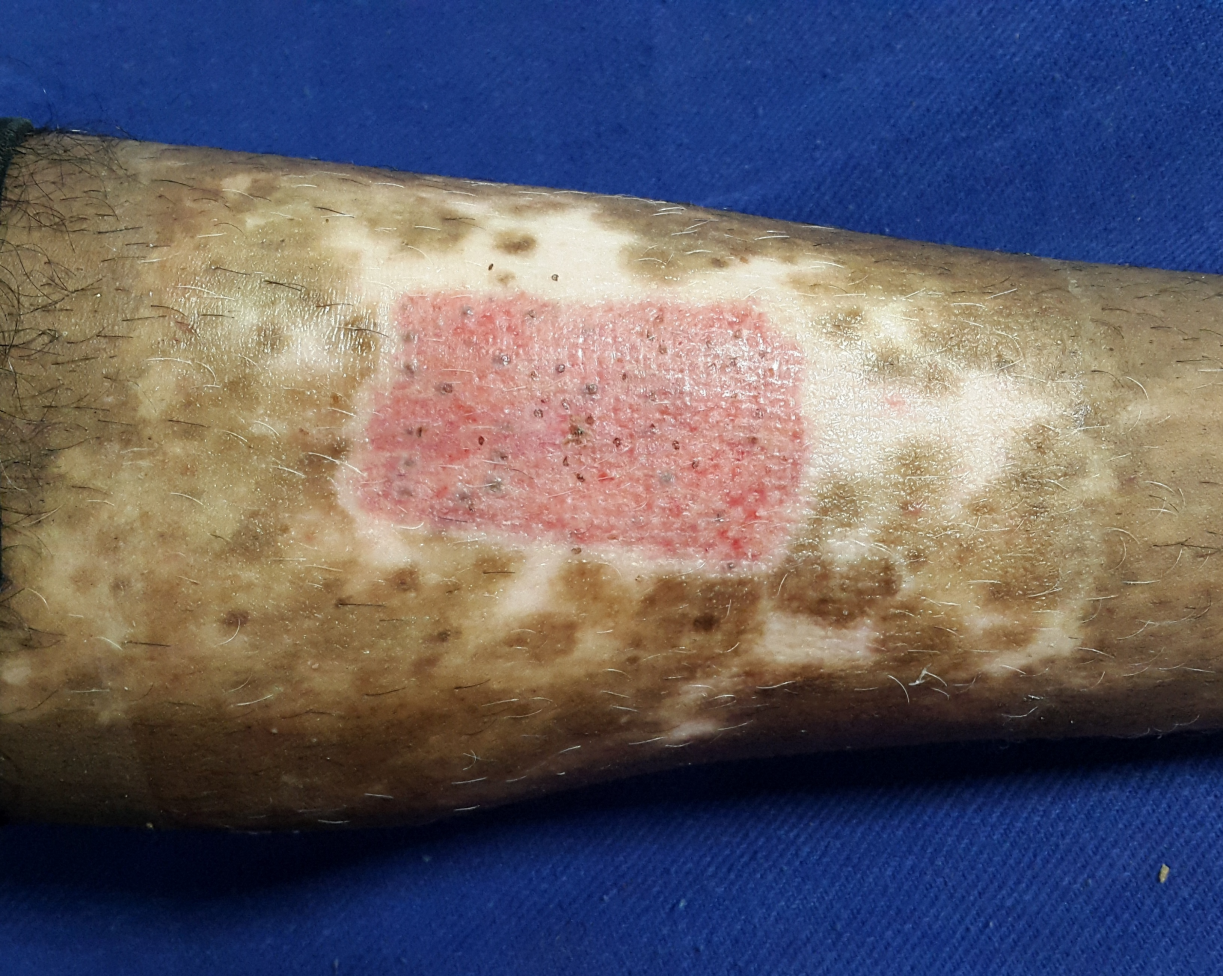
The recipient site at the end of two weeks showing uniform perigraft melanin pigment islands.

**Figure 10:**
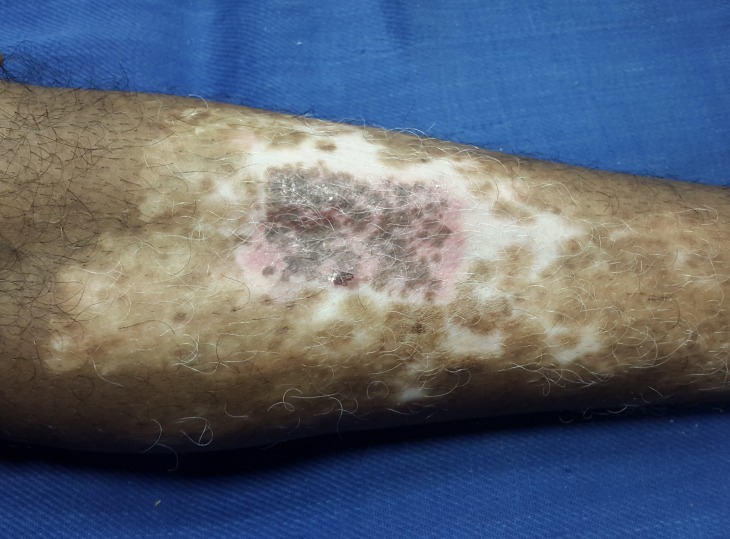
The recipient site at the end of four weeks showing 60-70% of repigmentation of the treated site.

**Figure 11:**
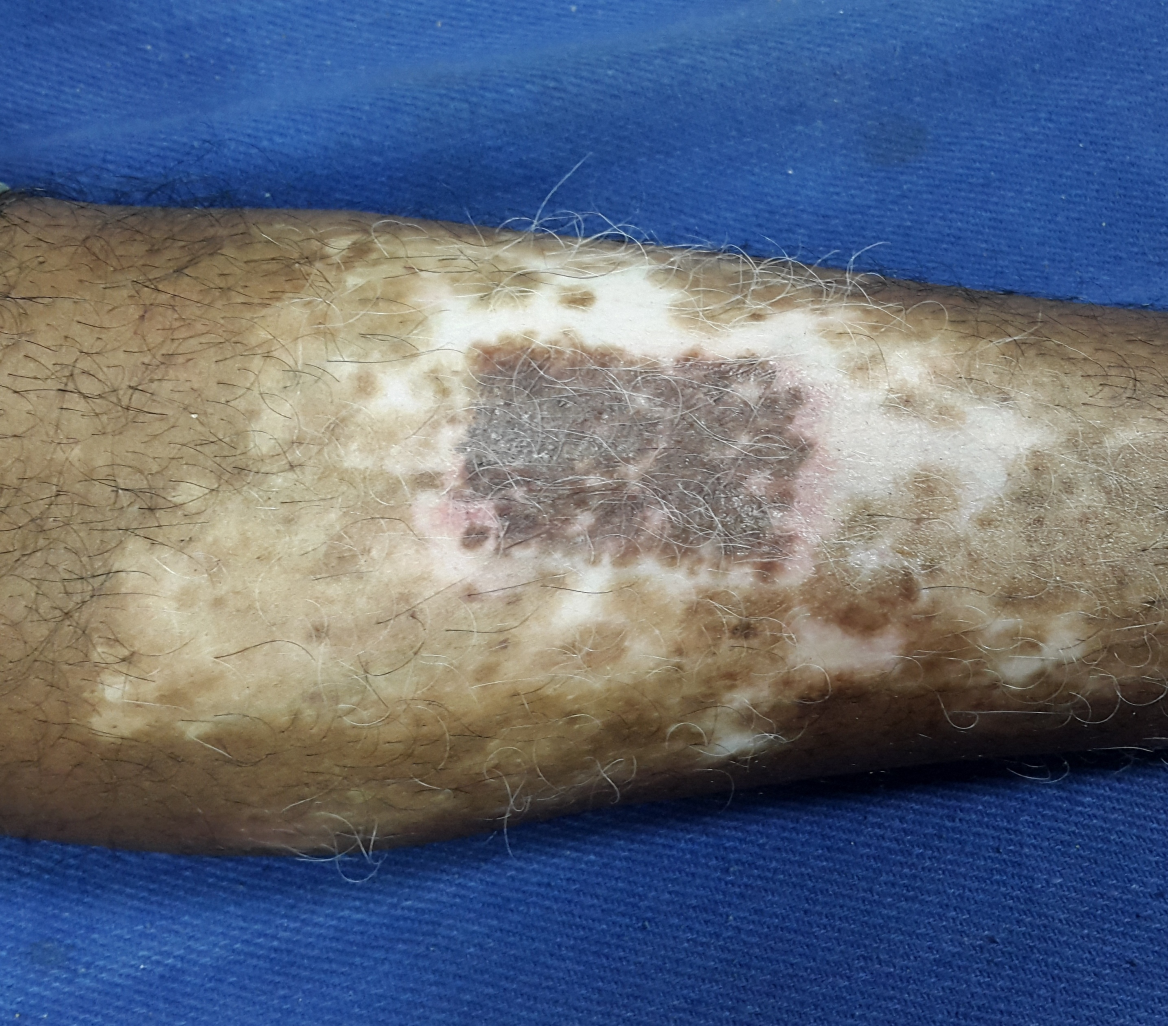
The recipient site at the end of eight weeks showing >95% of repigmentation of the treated site.

**Figure 12:**
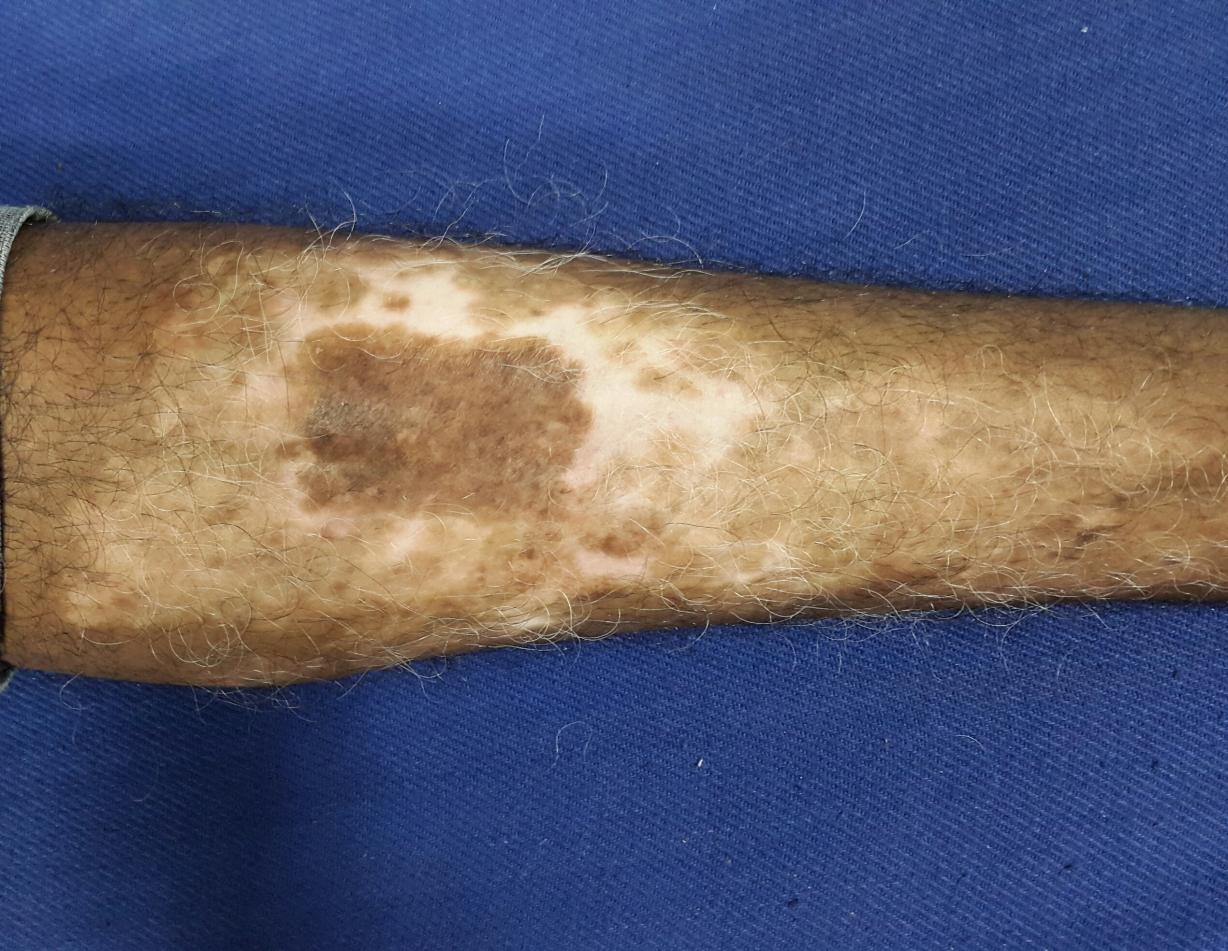
The recipient site at the end of 20 weeks showing uniform repigmentation of the treated site.

After a week, the dressing was removed carefully from both the donor and recipient sites. The donor site had epithelialized completely (Figure 7) and the recipient site showed most of the micrografts fallen off with scab formation, leaving behind pinkish areas of the donor grafts (Figure 8). The patient was followed-up every week and was assessed for the percentage of repigmentation and other complications like pain, secondary infection, cobble stoning, graft rejection, scarring, milia formation and reactivation of the disease, and the results were tabulated (Table 1). Photographs were taken on each follow-up for assessment. In the subsequent weekly follow-up uniform perigraft melanin pigment islands were observed (Figure 9) which gradually increased in size and coalesced to form 60-70% of repigmentation at the end of four weeks (Figure 10) and a near total repigmentation (>95%) at the end of eight weeks (Figure 11). At the end of 20 weeks, the pigmentation was uniform with no surface irregularities (Figure 12). No other topical medications were prescribed during this period. The patient’s quality of life was assessed using Dermatology Life Quality Index (DLQI) [3] once in four weeks. DLQI improved from a score of 20 to 9. The patient has been assessed for five months and will be on monthly follow-up for a period of 12 months and he is yet to receive more skin grafting for the surrounding vitiliginous patches using the negative pressure epidermal harvesting system.

**Table 1:** Table 1: Weekly assessment of the patient

No.of Weeks Postoperative	Percentage of Repigmentation	Erythema	Tenderness	Exudate	Cobble Stoning	Newer Vitiligo Lesions	Scarring/ Milia	Donor Site	DLQI
1	-	+	Nil	Nil	Nil	Nil	Nil	Healed completely	20
2	<10%	+	Nil	Nil	Nil	Nil	Nil	-	
3	30-40%	Nil	Nil	Nil	Nil	Nil	Nil	-	
4	60-70%	Nil	Nil	Nil	Nil	Nil	Nil	-	16
6	80-90%	Nil	Nil	Nil	Nil	Nil	Nil	-	
8	>95%	Nil	Nil	Nil	Nil	Nil	Nil	-	9
20	99%	Nil	Nil	Nil	Nil	Nil	Nil	-	7

## Discussion

Vitiligo is an acquired depigmentation disorder of great cosmetic importance affecting one–four percent of the world's population and one that has a major impact on the psychosocial life of the patients. There are many cases of vitiligo that either fail to or only partially respond to the medical line of treatment indicating that melanocyte reservoir [4] is no more available for repigmentation in these areas. Stability of vitiligo is manifested as the absence of new lesions, the absence of spread of existing vitiligo lesions, and absence of Koebner's phenomenon. Though the duration of stability is a debatable issue, the Indian Association of Dermatologists, Venereologists, and Leprologists (IADVL) task force has defined stability as 'a patient with no new lesions, no progression of existing lesions and absence of Koebner phenomenon during the past one year' [5]. In a recent study on the issue of vitiligo stability, a time period of 18 months of stable disease was shown to be the most suitable one for undertaking any grafting procedure [6]. Such patients with a stable disease respond well to surgical methods like tattooing, autologous skin grafting procedures like epidermal culture grafting, pure melanocyte culture grafting, epidermal grafting by suction blister technique, thin Thiersch's split skin grafting, or thin split skin miniature punch grafting. Proper selection of the patient is the most important factor for achieving a good cosmetic result with any grafting procedure in vitiligo. In our study, the disease was stable for two years.

The advantages of epidermal grafting over traditional split-thickness skin grafting include little or no patient discomfort during harvesting, obviating the need for anesthesia; minimal outpatient setting rather than an operating room; a superficial donor site wound that heals within two to four weeks with minimal scarring; and a simplified procedure that requires no surgical expertise. A novel automated, minimally invasive tool for generating autologous viable epidermal micrografts is now available commercially, which generates a negative pressure of -400 mm Hg to -500 mm Hg and heat of 37°C to 41°C to raise the epidermal microdomes in a harvester device, which are then adhered to a Tegaderm dressing and applied over the denuded recipient site. The epidermal microdomes formed at the derma-epidermal junction contain proliferative cells that secrete wound healing growth factors (e.g. vascular endothelial growth factor, transforming growth factor alpha, platelet-derived growth factor, hepatocyte growth factor, and granulocyte colony-stimulating factor) [7]. Hence the healing of the donor site wound is rapid and the repopulation of melanocytes at the recipient site is achieved faster compared to the other methods. There is also no risk of transmissible diseases as the harvester unit is disposable and the method is autologous. The entire procedure can be completed within 15 to 60 minutes, and there is no need for donor site anesthesia due to the absence of pain sensory organs in the epidermis, which results in minimal patient discomfort. The patients can return to their daily routine immediately, though they are asked to avoid strenuous activities for a week as the first 72 hours are most crucial for graft uptake due to fibrin bonding, and then begins the onset of vascular anastomosis and fibrovascular growth [8].

The common complications associated with other skin grafting procedures like cobble stoning, hyperpigmentation, perigraft halo, sinking pits, formation of milia and scarring, and beaded margins were not observed with the ANPEH method in our study. We observed a near total repigmentation in eight weeks which is way faster compared to other studies with epidermal grafting [9] where near total repigmentation took three to six months to appear. This method of ANPEH is already being used successfully to treat leg ulcers and chronic wounds. There are very minimal studies for the role of ANPEH in vitiligo.

## Conclusions

Hence the ANPEH technique serves as an ideal, simple, safe, faster, and cost-effective method of autologous epidermal harvesting in stable vitiligo. More controlled studies are required to statistically prove its effectiveness in this field.
